# Knowledge on hypertension among hypertensive stroke survivors in Ghana: A cross-sectional, multicenter study

**DOI:** 10.1016/j.neuros.2026.100060

**Published:** 2026-06-22

**Authors:** Rexford Adu Gyamfi, Albert Akpalu, Ansumana Bockarie, Priscilla Abrafi Opare-Addo, Nana Kwame Ayisi-Boateng, Douglas Anning Opoku, Jude Domfeh Darkwah, Emmanuel Konadu, Agnes Arthur, Kwadwo Agyenim-Boateng, Christiana Neizer, Timothy Fiattor, Nyantakyi Adu-Darko, Nathaniel Adusei Mensah, Raelle Tagge, Michael Ampofo, Hilda Kwapong, John Akassi, John Humphrey Amuasi, Samuel Blay Nguah, Bruce Ovbiagele, Fred Stephen Sarfo

**Affiliations:** Agogo Presbyterian Hospital, Asante Akyem, Ashanti Region, Ghana

**Keywords:** Knowledge, Hypertension, Stroke survivors, Sub-Saharan Africa

## Abstract

**Background:**

Hypertension is a premier risk factor for index and recurrent strokes globally. However, knowledge of hypertension as a risk factor for stroke occurrence and subsequent adverse vascular events among recent stroke survivors in the sub-Saharan Africa is limited.

**Purpose:**

To assess the level and determinants of knowledge on hypertension and risk of stroke among recent stroke survivors in Ghana.

**Methods:**

This is a cross-sectional survey conducted at enrollment of participants involved in the multi-center, phase III randomized trial titled Phone-based Intervention under Nurse Guidance after stroke II (PINGS-2) study. Recent stroke survivors within 1–2 months of stroke onset were recruited from 10 Ghanaian hospitals comprising of (five primary-level, two secondary-level, and three tertiary-level facilities) between 2020 and 2022. We assessed knowledge of hypertension and stroke using a validated 14-item questionnaire on hypertension and stroke. We analyzed data using univariate and multivariate analysis to determine the associations between sociodemographic variables and the knowledge on hypertension among recent stroke survivors.

**Results:**

We enrolled 500 participants, mean (SD) age of 58 (12) years, 56.2% being males and 73.3% had ischemic stroke. Out of 14 questions, the mean (SD) score was 8 (3) with a range of 0 to 13. Four factors significantly associated with knowledge of hypertension with adjusted *β* (95%CI) were Islam religion −1.40 (−2.40 - −0.49), NIHSS score −0.15 (− 0.22 - −0.07) for each unit increase and body mass index 0.08 (0.03 – 0.13) for each kg/m^2^ increase and educational attainment with primary level, secondary, and tertiary educational level 1.10 (0.02 – 2.10), 1.60 (0.52 – 2.70), 2.40 (1.20 – 3.60) compared with no education.

**Conclusion:**

We found substantial deficiencies in the knowledge on hypertension among recent hypertensive stroke survivors in the Ghanaian population in this multicenter study. Educational interventions are urgently needed to address these knowledge gaps to minimize risk of adverse outcomes among stroke survivors in resource-limited settings.

## 1. Introduction

Stroke is a leading cause of morbidity and the second leading cause of mortality across the globe [1,2]. The burden of stroke is more pronounced in lower-middle-income-countries (LMIC) compared to high income countries with approximately 90% of stroke morbidity and mortality occurring in LMIC, particularly in Sub-Saharan Africa (SSA) and Asia [3]. Stroke in LMICs afflicts a younger and productive age demographic with social, family, community, and national repercussions and threatens to hamper economy of developing countries.

In SSA, there are 1.4 adults per 1000 population living with stroke [4] who are at risk of further adverse cardiovascular events without appropriate secondary prevention interventions. Low levels of awareness on the modifiable vascular risk factors would inexorably exacerbate the risk for stroke recurrence [5] Undoubtedly, systemic arterial hypertension is a veritable risk factor for stroke recurrence globally. Sadly, a constellation of factors notably adverse socioeconomic status, low health literacy rates, and uncoordinated health systems in SSA have inordinately led to abysmally high blood pressure rates among hypertensives. Knowledge of stroke among hypertensive and diabetic patients is well documented [6–8], however, the determinants of the knowledge of hypertension and stroke among stroke survivors is limited in literature in developing countries.

To develop effective secondary prevention interventions aimed at improving outcomes of stroke survivors in LMICs, the level of knowledge of its risk factors and determinants of this knowledge is required. We therefore aimed to explore the determinants of knowledge of hypertension and stroke among recent stroke survivors in the Ghanaian population. The information generated will assist in developing targeted interventions to address the deficits in knowledge of hypertension and stroke among high-risk populations in resource-limited settings.

## 2. Methods

### 2.1. Study design and participants

This is a cross-sectional analysis of baseline data collected in a phase III randomized clinical trial titled Phone-based Intervention under Nurse Guidance after Stroke II study (PINGS-2). Briefly, PINGS 2 seeks to assess the efficacy of a phone-based, nurse-led, mobile health assisted intervention for blood pressure control among 500 recent Ghanaian stroke survivors recruited from ten study sites [9]. Study participants were adults (18 years or more) who had survived stroke after 1 to 2 months and were known to be hypertensive on antihypertensive medications after stroke. Ethical approval for the study was provided by the Committee on Human Publications and Ethics and all participants or their legal representatives provided informed consent before taking part in the study.

### 2.2. Measurements of determinants of hypertension and stroke knowledge

Trained Research Assistants provided a structured questionnaire to all the participants. Socio-economic and demographic variables collected included age, biological sex, location of residence, educational status, marital status, religion, and household monthly income. Clinical information collected includes stroke type (ischemic or intracerebral hemorrhage determined using head Computerized tomography), stroke severity at time of enrollment into the study using the National Institute of Health Stroke Scale (NIHSS) [9,10], functional status assessed using the Modified Rankin Score, and performance status using the Barthels Index [11–14]. Blood pressure at enrollment was measured using an automated blood pressure monitor in accordance with standardized clinical guidelines. Body mass index calculated after measuring body weight (in kg) and height (in meters).

### 2.3. Hypertension / stroke knowledge questionnaire

This is a 14-item questionnaire with questions on the following:

- cut-off values for defining hypertension (questions 1 and 2),
- knowledge on treatment of hypertension and duration of hypertension treatment (questions 3 and 4),
- lifestyle factors that may affect blood pressure control (questions 5 and 6),
- deleterious effects of hypertension on health (question 7 to 10),
- risk of stroke recurrence (questions 11 and 14) and adherence to antihypertensive medications (questions 12 and 13)

### 2.4. Statistical analysis

Baseline demographic, clinical characteristics, and responses to hypertension/stroke knowledge questionnaire of study participants were presented as frequencies and percentages. The median score on the HKSQ was used to dichotomize the study participants for comparison. Means and medians of continuous variables were compared using Students *t*-tests or the Wilcoxon rank sum test respectively. Proportions were compared using the Pearson’s Chi-square test or Fisher’s exact test. Next, a multivariate linear regression model was constructed to identify factors independently associated with scores obtained on the HSKQ. The independent variables included in the model were age, gender, marital status, educational status, living status, religion, domicile, monthly income, stroke type (ischemic, hemorrhagic, ischemic with hemorrhage), etiological subtypes of stroke, body mass index (BMI) and Barthels index. Throughout the analysis, p-value cut-off < 0.05 was deemed statistically significant.

## 3. Results

### 3.1. Demographic and clinical characteristics of participants

Of the 500 participants recruited, 470 (94%) of the study participants completed the HSKQ. The age range for the participants was from 24 to 88 years with a mean (±SD) age was 58 ± 12 years. There were 281 (56.2%) males. The proportion of participants with ischemic stroke was 306 (61.2%) while 109 (21.8%) had hemorrhagic stroke and 85 (17%) had missing information on stroke type. (Table 1)

Of the participants, 142 (30.2%) received their health care from a primary facility while 111 (23.6%) and 217 (46.2%) received care from secondary and tertiary care levels respectively. The place of domicile for a greater proportion of the participants was in the urban area followed by semi-urban and rural in the proportions of 279 (59.4%), 157 (33.4%) and 35 (7.2%) respectively. Regarding educational level, 44 (9.4%) had no formal education while 193 (41.1%), 156 (33.2%) and 77 (16.4%) had primary, secondary and tertiary levels of education respectively. Table one describes the sociodemographic and clinical characteristics of the recent stroke survivors.

### 3.2. Knowledge on hypertension and stroke among participants

Only 24% of respondents knew that a blood pressure of 115 / 75 mmHg was normal while up to 67% correctly identified a BP of 160 / 100 mmHg as elevated and 36% knew hypertension was a chronic medical condition. (Table 2). Regarding the control of hypertension, 95% knew that antihypertensive medications were to be taken every day, 66% and 75% respectively responded that weight loss and eating less salt would lead to lower BP. On the deleterious effects of hypertension, 70%, 48%, and 79% correctly associated hypertension with heart attacks, kidney problems, and stroke respectively.

### 3.3. Demographic and clinical characteristics of participants by questionnaire scores

Out of 14 questions, the mean (SD) score was 8 (3) with a range of 0 to 13. Participants whose scores were above the median were more likely to be male, had higher educational status with higher monthly income (Table 3). Clinically, those whose scores were above the median had a significantly lower stroke severity on the NIHSS but no differences in stroke type and blood pressures were observed.

### 3.4. Factors associated with hypertension and stroke knowledge

On bivariate linear regression, we identified that male sex, educational status, religion, modified Rankin score, NIH stroke scale score, body mass index and level of health institution were associated with scores obtained on the knowledge questionnaire (Table 4). Four factors significantly associated with knowledge of hypertension with adjusted (95%CI) were islam religion −1.40 (−2.40 - −0.49), NIHSS score −0.15 (− 0.22 - −0.07) for each unit increase and body mass index 0.08 (0.03 – 0.13) for each kg/m^2^ increase and educational attainment with primary level, secondary, and tertiary educational level 1.10 (0.02 – 2.10), 1.60 (0.52 – 2.70), 2.40 (1.20 – 3.60) compared with no education.

## 4. Discussion

In this multicenter study in Ghana, we have identified deficiencies in the knowledge on hypertension, the premier risk factor for stroke occurrence and recurrence worldwide. The four factors significantly associated with knowledge of hypertension were educational status, islam religion, body mass index and stroke severity.

In unadjusted analyses, we found that male sex is a strong determinant of the knowledge of hypertension and stroke after stroke occurrence. This finding was comparable to two previous studies in Asia (Malaysia and China) which investigated gender differences and the associations between hypertension and stroke knowledge [15, 16]. Whiles the Malaysian study found male sex to be associated with higher knowledge of hypertension and stroke and that the males knew what to do during stroke [15], the Chinese study found that although male patients had greater stroke knowledge, they exhibited worse pre-stroke health behaviors [16]. These findings were also corroborated by findings from the Stroke Investigative Research and Educational Network (SIREN) study which found that males usually present with less severe forms of stroke than females in Africa [17] which could be attributable to the higher level of knowledge of hypertension and stroke among males. The poor knowledge of stroke among females may always explain the higher incidence of strokes among female [18–21]. This is a clarion call for intensification of stroke education with great emphasis on its risk factors and warning signs through public or social media and health education targeting the adult populations with a special focus on the females because approximately 80% of new stroke incidence could be avoided by improving knowledge related to the risk factors of stroke [22].

We found graded association between the level of education and the knowledge of hypertension. The higher the level of education, the higher the knowledge of hypertension and stroke. Tertiary level education was the strongest determinant of one’s level of knowledge of hypertension and stroke followed by secondary and primary levels. This finding was comparable with several published data in both the developing and the developed countries. In Nepal, South Asia, Ghimire et al. found that higher level of education was significantly associated with good level of knowledge of hypertension and stroke [23]. Similarly, a longitudinal data comparing 10 European populations demonstrated that stroke mortality is negatively affected by lower levels of education, with similar effects on both males and females with relative risk of 1.27 (95% CI, 1.24 to 1.30) in men and relative risk of 1.29 (95% CI, 1.27–1.32) in women [24], thus, higher education empowers the individual to master the vascular risk factors of stroke as well as the signs and symptoms of stroke so as to put in the necessary interventions to prevent the occurrence of an index stroke or to adhere strictly to secondary preventive measures after stroke. Also, poor level of education has a greater influence on the individual’s health seeking behavior. Studies from Ghana and Nigeria have shown that lower educational level and lower socioeconomic status, are paramount among the reasons why sick persons in some rural communities in Africa will not seek orthodox medical care as the first option [25,26].

Our study found a very significant association between the National Institute of Health Stroke Scale (NIHSS) score and the knowledge of hypertension and stroke with a P-value of (<0.001). There was paucity of data on this subject, thereby making it difficult to directly compare our findings to previously published data, however, significant inferences could be made. Kabanda et al. found that, in Kinshasa, Democratic Republic of Congo, patients with lower NIHSS were less likely to report early to the hospital [27]. A similar report was found in India where Ashraf et al., reported that patients with higher NIHSS score report early to the hospital after stroke occurence [28]. Since patients with severe forms of stroke present with higher NIHSS score [29], the patients or their caregivers can recognize that stroke might have occurred and report early for interventions. Another plausible explanation to this phenomenon is the fact that the poor knowledge of hypertension and stroke as alluded to by our study and has been confirmed by other studies [15–17] could be the reason why women usually present with severe forms of stroke [29–31].

We observed in this current study that, among recent stroke survivors, one’s religious affiliation is associated with the level of knowledge of hypertension and stroke. Being Islam was significantly associated with lower level of knowledge of hypertension and stroke with a beta estimation value of (−1.4) 95% C.I (−2.4–0.49) and a P-value of (0.003). There is a dearth of evidence regarding this matter in literature, however, a study from rural Tanzania has shown a gross misconception among religious leaders regarding the knowledge of hypertension [32]. The perception about hypertension by some of the Islamic religious leaders in rural Tanzania is akin to the findings in this current study and points us in the right direction as where to focus our energies in our bid to control the stroke epidemic in Africa. Religious misconceptions about the knowledge of hypertension, the most robust modifiable risk factor for stroke is widespread in Africa with evidence from Ghana, Nigeria, Democratic Republic of Congo, and Uganda [33–36]. Since religious leaders have great influence on their followers, it will be quite appropriate to empower them with the right information about hypertension and stroke and leverage on their influence to reduce the burden of hypertension and stroke in a continent where greater proportion of the people reports highest confidence in religious institutions (76%) as against 51% in health institutions and 44% in government [37].

The current study found a significant relationship between Body Mass Index (BMI) and the knowledge of hypertension and stroke among recent stroke survivors with a beta estimate of (0.08), 95% CI of (0.03–0.13) and a P-value of (0.002). The higher one’s BMI, the higher their level of knowledge of hypertension and stroke. Comparable data assessing the relationship between BMI and the knowledge of hypertension and stroke among stroke survivors was rare in literature. However, data from Ghana and Venezuela assessing same among healthy individuals without stroke found no significant association between BMI and the knowledge of hypertension and stroke [38,39]. The differences could be attributable to the patient category in the different studies. The strong association between high BMI and the incidence of stroke should be a motivating factor for clinicians to spend additional time educating obese patients on the cardiovascular complications of obesity. Public health agencies must also be interested in intensifying the education of obesity and its complications.

## 5. Conclusions

As observed in this present study, there are significant gaps in the knowledge on hypertension and stroke among recent stroke survivors in the Ghanaian population. Overall, factors that determined the knowledge on hypertension and risk of stroke recurrence among recent stroke survivors in Ghana includes male sex, higher level of education and high BMI. These findings support the need to intensify the education on hypertension and stroke to beat the stroke epidemics in Ghana.

## Figures and Tables

**Table 1:** Demographic and clinical characteristics of study participants.

Characteristic	N = 500^1^
**Age in years**	
Median (IQR)	58 (51, 67)
Mean (SD)	58 (12)
Range	24, 88
**Gender**	
Male	281 (56.2)
Female	219 (43.8)
**Marital Status**	
Currently Married	333 (66.6)
Previously Married	144 (28.8)
Never Married	23 (4.6)
**Educational Status**	
None	49 (9.8)
Primary	205 (41.0)
Secondary	164 (32.8)
Tertiary	82 (16.4)
**Living Status**	
Lives Alone	29 (5.8)
Lives With Spouse and Children	272 (54.4)
Lives in a Nursing Home	1 (0.2)
Lives With Spouse	30 (6.0)
Lives With Extended Family	72 (14.4)
Lives With Children	96 (19.2)
**Religion**	
Christianity	448 (89.6)
Islam	49 (9.8)
Other	3 (0.6)
**Domicile**	
Rural	35 (7.0)
Semi-Urban	165 (33.0)
Urban	300 (60.0)
**Income in GHC**	
0–100	102 (20.6)
101–250	160 (32.4)
251–500	145 (29.4)
>500	87 (17.6)
Missing	6
**Stroke Type (Choose One)**	
Ischemic Stroke	306 (61.2)
Intracerebral Hemorrhagic Stroke	109 (21.8)
Untyped Stroke (no CT scan available)	85 (17)
**Body Mass Index**	
Median (IQR)	26.2 (22.7, 30.0)
Mean (SD)	26.6 (5.5)
Range	11.4, 47.9
Missing	24
**Barthels Index**	
Median (IQR)	80 (40, 90)
Mean (SD)	66 (27)
Range	0, 90
Missing	19
**Health institution category**	
Primary	148 (29.6)
Secondary	119 (23.8)
Tertiary	233 (46.6)

**Table 2:** Item by item response on the hypertension / stroke knowledge questionnaire by participants.

QUESTION AND ANSWER OPTIONS	RESPONSE RATE N = 500^1^
**(1) If someone’s blood pressure is 115/75. it is ……**	
High	51 (10.3)
Low	158 (31.8)
Normal	119 (23.9)
Don’t Know	169 (34.0)
Missing	3
**(2) If someone’s blood pressure is 160/100. It is.…**	
High	330 (66.7)
Low	10 (2.0)
Normal	9 (1.8)
Don’t Know	146 (29.5)
Missing	5
**(3) Once someone has high blood pressure, it usually lasts for**	
A few years	76 (15.4)
5–10 Years	25 (5.1)
The Rest of their Life	178 (36.0)
Don’t Know	216 (43.6)
Missing	5
**(4) People with high blood pressure should take their medicine**	
Everyday	465 (95.3)
At Least a few Times a week	11 (2.3)
Only When They feel sick	12 (2.5)
Missing	12
**(5) Losing weight usually makes blood pressure**	
Go up	40 (8.2)
Go Down	322 (66.4)
Stay the same	123 (25.4)
Missing	15
**(6) Eating less salt usually makes blood pressure**	
Go Up	50 (10.2)
Go Down	370 (75.2)
Stay the Same	72 (14.6)
Missing	8
**(7) High blood pressure can cause heart attacks**	
Yes	345 (69.7)
No	11 (2.2)
Don’t Know	139 (28.1)
Missing	5
**(8) High blood pressure can cause cancer**	
Yes	147 (29.6)
No	59 (11.9)
Don’t Know	290 (58.5)
Missing	4
**(9) High blood pressure can cause can kidney problems**	
Yes	238 (48.0)
No	23 (4.6)
Don’t Know	235 (47.4)
Missing	4
**(10) High blood pressure can cause strokes**	
Yes	392 (79.2)
No	8 (1.6)
Don’t Know	95 (19.2)
Missing	5
**(11) Someone who has had a stroke is at higher risk of having another**	
Yes	258 (52.1)
No	29 (5.9)
Don’t Know	208 (42.0)
Missing	5
**(12) If someone is not having headaches they can stop taking medications**	
Yes	94 (19.0)
No	274 (55.4)
Don’t Know	127 (25.7)
Missing	5
**(13) If someone is feeling good it is ok to miss doses of medication**	
Never	354 (71.5)
Once a Month	11 (2.2)
Once a week	7 (1.4)
Don’t know	123 (24.8)
Missing	5
**(14) Once someone has had a stroke, they will be at risk for stroke for ….**	
A Few Years	139 (28.2)
5–10 Years	21 (4.3)
The Rest of Their Life	62 (12.6)
Don’t Know	271 (55.0)
Missing	7
**Total HKQ Score**	
Median (IQR)	8 (6, 10)
Mean (SD)	8 (3)
Range	0, 13
Missing	30
**Categorised Total HKQ Score**	
**Median & below**	240 (51.1)
Above Median	230 (48.9)
Missing	30

**Table 3:** Comparison of demographic and clinical characteristics of study participants by their median score on HKQ.

Characteristic	Categorised Total HKQ Score		Overall, N = 470^1^	p-value^2^
	Median & below, N = 240^1^	Above Median, N = 230^1^		
**Age in years**	58 (50, 68)	58 (52, 65)	58 (51, 67)	0.717
**Male sex**	115 (47.9)	144 (62.6)	259 (55.1)	0.001
**Educational Status**				<0.001
None	32 (13.3)	12 (5.2)	44 (9.4)	
Primary	117 (48.8)	76 (33.0)	193 (41.1)	
Secondary	71 (29.6)	85 (37.0)	156 (33.2)	
Tertiary	20 (8.3)	57 (24.8)	77 (16.4)	
**Religion**				0.060
Christianity	208 (86.7)	212 (92.2)	420 (89.4)	
Islam	29 (12.1)	18 (7.8)	47 (10.0)	
Other	3 (1.3)	0 (0.0)	3 (0.6)	
**Domicile**				0.376
Rural	14 (5.8)	20 (8.7)	34 (7.2)	
Semi-Urban	85 (35.4)	72 (31.3)	157 (33.4)	
Urban	141 (58.8)	138 (60.0)	279 (59.4)	
**Income in GHC**				0.001
0–100	109 (45.6)	58 (25.2)	167 (35.5)	
101–250	64 (26.6)	79 (34.3)	143 (30.4)	
251–500	43 (17.7)	61 (26.5)	104 (22.1)	
>500	24 (10.1)	32 (13.9)	56 (12.0)	
**Stroke Type (Choose One)**				0.055
Ischemic Stroke	154 (64.7)	169 (72.8)	323 (68.7)	
Intracerebral	50 (21.0)	45 (19.4)	95 (20.2)	
Hemorrhagic Stroke				
Untyped Stroke (no CT scan available)	34 (14.3)	18 (7.8)	52 (11.1)	
**Modified Rankin Score**				0.023
Median (IQR)	2.00 (1.00, 3.00)	2.00 (1.00, 3.00)	2.00 (1.00, 3.00)	
**NIH Stroke Scale**	4.0 (0.0, 9.0)	2.0 (0.0, 6.0)	3.0 (0.0, 8.0)	<0.001
**Systolic blood pressure (mm Hg)-Baseline**	155 (146, 172)	154 (146, 168)	155 (146, 170)	0.265
**Diastolic Blood Pressure**	96 (89, 106)	94 (87, 103)	95 (88, 105)	0.070
**Body Mass Index**	25.9 (22.4, 28.7)	26.4 (23.0, 30.9)	26.2 (22.7, 30.1)	0.059
Health institution category				0.672
Primary	75 (31.3)	67 (29.1)	142 (30.2)	
Secondary	59 (24.6)	52 (22.6)	111 (23.6)	
Tertiary	106 (44.2)	111 (48.3)	6.2)	

1 Median (IQR); n (%).

2 Wilcoxon rank sum test; Pearson’s Chi-squared test; Fisher’s exact test.

**Table 4:** Factors associated with hypertension knowledge among recent Ghanaian stroke survivors.

VARIABLE	BIVARIATE			MULTIVARIATE	
		Unadjusted *β* (95% CI)	P-value	Adjusted *β* (95% CI)	P-value
**Age in years**	470	−0.02 (−0.05, 0.00)	0.092		
Male sex	470				
No		Ref		—	
Yes		0.67 (0.10, 1.20)	**0.022**	0.41 (−0.21, 1.00)	0.196
**Educational Status**	470				
None		Ref		Ref	
Primary		1.50 (0.51, 2.50)	**0.003**	1.10 (0.02, 2.10)	**0.046**
Secondary		2.20 (1.20, 3.20)	**<0.001**	1.60 (0.52, 2.70)	**0.004**
Tertiary		3.10 (2.00, 4.30)	**<0.001**	2.40 (1.20, 3.60)	**<0.001**
**Religion**	470				
Christianity		Ref		Ref	
Islam		−1.6 (−2.6, −0.69)	**<0.001**	−1.4 (−2.4, −0.49)	**0.003**
Other		−3.6 (−7.1, −0.04)	**0.048**	−3.4 (−7.5, 0.68)	0.102
**Domicile**	470				
Rural		Ref			
Semi-Urban		−0.87 (−2.0, 0.30)	0.143		
Urban		−0.94(−2.1,0.18)	0.101		
**Monthly**	470				
0–100		0.04(−0.77,−0.84)	0.930		
101–250		0.32(−0.51,−1.1)	0.451		
251–500		0.32 (−0.51, 1.1)	0.451		
>500		0.31 (−0.63, 1.3)	0.512		
**Stroke Type (Choose One)**	405				
Ischemic Stroke		Ref			
Intracerebral Hemorrhagic Stroke		−0.48 (−1.2, 0.21)	0.170		
Ischemic With Hemorrhagic Transformation		−0.40 (−2.4, 1.6)	0.692		
Untyped Stroke		−1.9 (−4.5, 0.76)	0.163		
**Modified Rankin Score**	469	−0.39 (−0.61, −0.16)	**<0.001**	0.05 (−0.25, 0.35)	0.726
**NIH Stroke Scale**	461	−0.13 (−0.18, −0.08)	**<0.001**	−0.15 (−0.22, −0.07)	**<0.001**
**Systolic blood pressure (mm Hg)-Baseline**	467	−0.01 (−0.03, 0.00)	0.113		
**Diastolic Blood Pressure**	467	−0.01 (−0.03, 0.01)	0.216		
**Body Mass Index**	451	0.10 (0.05, 0.15)	**<0.001**	0.08 (0.03, 0.13)	**0.002**
**Health institution category**	470				
Primary		Ref		Ref	
Secondary		0.81 (0.03, 1.60)	**0.042**	0.48 (−0.30, 1.30)	0.228
Tertiary		0.50 (−0.16, 1.2)	0.137	−0.39 (−1.1, 0.34)	0.297

C.I Confidence Interval.

## References

[1] World Stroke Campaign. WSD2022_Campaign_Toolkit_LR.pdf. https://www.world-stroke.org/world-stroke-day-campaign/world-stroke-campaign

[2] Global Stroke Factsheet. Int J Stroke 2022;17(1):18–29. 34986727 10.1177/17474930211065917

[3] Owolabi MO, Akarolo-Anthony S, Akinyemi R, Arnett D, Gebregziabher M, Jenkins C, The burden of stroke in Africa: a glance at the present and a glimpse into the future. Cardiovasc J Afr 2015;26(2 Suppl 1):S27–38 PMID: 25962945; PMCID: PMC4557491. doi:10.5830/CVJA-2015-038. 25962945 PMC4557491

[4] Adebayo O, Akpa O, Asowata OJF, Adekunle S, Akpalu FS, Albert, Determinants of first-ever stroke severity in West Africans: evidence from the SIREN study. J Am Heart Assoc 2023;12. doi:10.1161/JAHA.122.027888. PMC10356032 37301737

[5] The rising global burden of stroke. J eClin Med 2023;59:102028. doi:10.1016/j.eclinm.2023.102028. PMC10225620 37256100

[6] Nepal GM, Chand P, Poudel K, Acharya SR. Knowledge of stroke among hypertensive patients in Dhulikhel. Kathmandu Univ Med J 2022;78(3):141–6. 37017156

[7] Tuli W, Teshome E, Jiru T. Knowledge of stroke risk factors and prevention among hypertensive patients on follow-up at Addis Ababa University Tertiary Hospital, Addis Ababa, Ethiopia: a cross-sectional study. BMJ Open 2024;14:e089159. doi:10.1136/bmjopen-2024-089159. PMC11580316 39572087

[8] Mosenzon O, Cheng AY, Rabinstein AA, Sacco S. Diabetes and stroke: what are the connections? J Stroke 2023;25(1):26–38 Epub 2023 Jan 3. PMID: 36592968; PMCID: PMC9911852. doi:10.5853/jos.2022.02306. 36592968 PMC9911852

[9] Adams HP, Davis PH, Leira EC, Chang KC, Bendixen BH, Clarke WR, Baseline NIH Stroke Scale score strongly predicts outcome after stroke. Neurol [Internet] 1999;53(1). Jul 1 [cited 2023 Nov 29]126–126Available from: http://n.neurology.org/content/53/1/126. 10.1212/wnl.53.1.126 10408548

[10] Spilker J, Kongable G, Barch C, Braimah J, Bratina P, Daley S, Using the NIH stroke scale to assess stroke patients. J Neurosci Nurs [Internet] 1997;29(6):384–92. [cited 2023 Nov 29]Available from: https://go.gale.com/ps/i.do?p=AONE&sw=w&issn=08880395&v=2.1&it=r&id=GALE%7CA20329809&sid=googleScholar&linkaccess=fulltext. 9479660 10.1097/01376517-199712000-00008

[11] Nobels-Janssen E, Postma EN, Abma IL, van Dijk JMC, Haeren R, Schenck H, Inter-method reliability of the modified Rankin Scale in patients with subarachnoid hemorrhage. J Neurol [Internet] 2022;269(5):2734–42. [cited 2023 Nov 29]Available from: https://link.springer.com/article/10.1007/s00415-021-10880-4. 34746964 10.1007/s00415-021-10880-4 PMC8572691

[12] Sulter G, Steen C, De Keyser J. Use of the Barthel index and modified Rankin scale in acute stroke trials. Stroke 1999;30(8):1538–41 PMID 10436097. doi:10.1161/01.str.30.8.1538. 10436097

[13] O’Sullivan SB, Schmitz TJ. Physical rehabilitation. Philadelphia, PA: F.A. Davis Company; 2007. p. 385.

[14] Carroll D “Functional Evaluation: the Barthel Index” *(PDF)*. Archived from the original *(PDF)* on 28 September 2011. Retrieved 12 May 2011.

[15] Ching SM1, Chia YC2, Navin Kumar1 D, Lim HM3, Beh HC3. Ps-P06-6: gender differences on determinants of good knowledge on recognition of stroke symptoms and actions during a stroke among hypertensive patient. J. Hypertens 2023;41(Suppl 1):e254. doi:10.1097/01.hjh.0000915312.80364.98.

[16] Li ZR, Ruan HF, Shen LP, Zhang XP, Wan LH. Gender difference in the association between stroke knowledge and health behavior before the onset of stroke among Chinese hypertensive patients. J Neurosci Nurs 2021;53(4):160–5 PMID: 34116556. doi:10.1097/JNN.0000000000000599. 34116556

[17] Akpalu A, Gebregziabher M, Ovbiagele B, Sarfo F, Iheonye HA, Rufus A, On behalf of SIREN Team as part of H3Africa Consortium, differential impact of risk factors on stroke occurrence among men versus women in West Africa. J Am Heart Assoc 2019;50:820–7. doi:10.1161/STROKEAHA.118.022786. PMC6433514 30879432

[18] Heron M Deaths: leading causes for 2019. Natl Vital Stat Rep 2021;70:1–114. 34520342

[19] Virani SS, Alonso A, Aparicio HJ, Benjamin EJ, Bittencourt MS, Callaway CW, Carson AP, Chamberlain AM, Cheng S, Delling FN, American heart association council on epidemiology and prevention statistics committee and stroke statistics subcommittee. Heart disease and stroke statistics-2021 update: a report from the American heart association. Circulation. 2021;143:e254–743. doi:10.1161/CIR.0000000000000950. 33501848 PMC13036842

[20] Madsen TE, Khoury JC, Leppert M, Alwell K, Moomaw CJ, Sucharew H, Woo D, Ferioli S, Martini S, Adeoye O, Temporal trends in stroke incidence over time by sex and age in the GCNKSS. Stroke 2020;51:1070–6. doi:10.1161/strokeaha.120.028910. 32078459 PMC7286565

[21] Rehman S, Sahle BW, Chandra RV, Dwyer M, Thrift AG, Callisaya M, Breslin M, Phan HT, Otahal P, Gall S. Sex differences in risk factors for aneurysmal subarachnoid haemorrhage: systematic review and meta-analysis. J Neurol Sci 2019;406:116446. doi:10.1016/j.jns.2019.116446. 31521957

[22] Dar NZ, Khan SA, Ahmad A, Maqsood S. Awareness of stroke and health-seeking practices among hypertensive patients in a tertiary care hospital: a cross-sectional survey. Cureus 2019;11(5):e4774. doi:10.7759/cureus.4774. 31367493 PMC6666879

[23] Ghimire S, Shrestha S, Thapa L. Factors influencing stroke awareness among patients with hypertension in a tertiary hospital of Nepal: a cross-sectional study (P6. 003). AAN Enterprises 2018.

[24] Avendano M, Kunst AE, Huisman M, van Lenthe F, Bopp M, Borrell C, Educational level and stroke mortality. A comparison of 10 European populations during the 1990s. Stroke 2004;35:432–7. 14726555 10.1161/01.STR.0000109225.11509.EE

[25] Kuuire VZ, Bisung E, Rishworth A, Dixon J, Luginaah I. Health-seeking behaviour during times of illness: a study among adults in a resource poor setting in Ghana. J Public Health (Bangkok) 2016;38(4):fdv176 Pages. doi:10.1093/pubmed/fdv176. 28158717

[26] Latunji OO, Akinyemi oo. Factors influencing health seeking beheavior among civil servants in Ibadan, Nigeria. Ann Ib Postgrad med 2018(1):52–60. 30254559 PMC6143883

[27] Igor KK, Credo KN, Christian-Khalifa EB, Jean-Paul DN, Nono KP, Olivier TM, Stroke signs knowledge and factors associated with a delayed hospital arrival of patients with acute stroke in Kinshasa. Heliyon 2024;10(Issue 7):e28311 ISSN 24058440. doi:10.1016/j.heliyon.2024.e28311. 38571603 PMC10988012

[28] Ashraf VV, Factors delaying hospital arrival of patients with acute stroke. Ann. Indian Acad. Neurol 2015;18(2):162. 26019412 10.4103/0972-2327.150627 PMC4445190

[29] Fredwall M, Sternberg S, Blackhurst D, Lee A, Leacock R, Nathaniel TI. Gender differences in exclusion criteria for recombinant tissue-type plasminogen activator. J Stroke Cerebrovasc Dis 2016;25(11):2569–74 [PubMed] [Google Scholar]. 27618196 10.1016/j.jstrokecerebrovasdis.2016.06.012

[30] Blum B, Wormack L, Holtel M, Penwell A, Lari S, Walker B, Gender and thrombolysis therapy in stroke patients with incidence of dyslipidemia. BMC Womens Health 2019:11 [PMC free article] [PubMed] [Google Scholar]. 10.1186/s12905-018-0698-6 PMC6335821 30651099

[31] Branyan TE, Sohrabji F. Sex differences in stroke co-morbidities. Exp Neurol 2020;332:113384 [PMC free article] [PubMed] [Google Scholar]. 32585156 10.1016/j.expneurol.2020.113384 PMC7418167

[32] Lambert VJ, Kisigo GA, Nzali A, Laizer E, Paul N, Walshe L, Religious leaders as trusted messengers in combatting hypertension in rural Tanzanian communities. Am J Hypertens 2021;34(10):1042–8 Published online 2021 May 22PMCID: PMC9214778. doi:10.1093/ajh/hpab080. 34022044 PMC9214778

[33] Odusola AO, Hendriks M, Schultsz C, Bolarinwa OA, Akande T, Osibogun A, Agyemang C, Ogedegbe G, Agbede K, Adenusi P, Lange J, van Weert H, Stronks K, Haafkens JA. Perceptions of inhibitors and facilitators for adhering to hypertension treatment among insured patients in rural Nigeria: a qualitative study. BMC Health Serv Res 2014;14:624 [PMC free article] [PubMed] [Google Scholar]. 25491509 10.1186/s12913-014-0624-z PMC4267751

[34] Nyaaba GN, Masana L, Aikins AD, Stronks K, Agyemang C. Lay community perceptions and treatment options for hypertension in rural northern Ghana: a qualitative analysis. BMJ Open 2018;8:e023451 [PMC free article] [PubMed] [Google Scholar]. 10.1136/bmjopen-2018-023451 PMC6278795 30498042

[35] Cremers AL, Alege A, Nelissen HE, Okwor TJ, Osibogun A, Gerrets R, Van’t Hoog AH. Patients’ and healthcare providers’ perceptions and practices regarding hypertension, pharmacy-based care, and mHealth in Lagos, Nigeria: a mixed methods study. J Hypertens 2019;37:389–97 [PMC free article] [PubMed] [Google Scholar]. 30645210 10.1097/HJH.0000000000001877 PMC6365248

[36] Lulebo AM, Mapatano MA, Mutombo PB, Mafuta EM, Samba G, Coppieters Y. Prevalence and determinants of use of complementary and alternative medicine by hypertensive patients attending primary health care facilities in Kinshasa, Democratic Republic of the Congo: a cross-sectional study. BMC Complement Altern Med 2017;17:205 [PMC free article] [PubMed] [Google Scholar]. 28390416 10.1186/s12906-017-1722-3 PMC5385009

[37] Tortora B Africans’ confidence in institutions—Which country stands out? Gallup News Serv 2007. https://news.gallup.com/poll/26176/africans-confidence-institutions-which-country-stands-out.aspx [Google Scholar].

[38] Asante DO, Dai A, Walker AN, Zhou Z, Kpogo SA, Lu R, Huang K, Zou J. Assessing hypertension and diabetes knowledge, attitudes and practices among residents in Akatsi South District, Ghana using the KAP questionnaire. Front Public Health 2023;11:1056999 PMID: 37333544; PMCID: PMC10272529. doi:10.3389/fpubh.2023.1056999. 37333544 PMC10272529

[39] Lugo-Mata ÁR, Urich-Landeta AS, Andrades-Pérez AL, León-Dugarte MJ, Marcano-Acevedo LA, Jofreed López Guillen MH , Factors associated with the level of knowledge about hypertension in primary care patients, Medicina Universitaria, 16655796 DOI: 10.1016/j.rmu.2017.10.008

